# Trends in absolute socioeconomic inequalities in mortality in Sweden and New Zealand. A 20-year gender perspective

**DOI:** 10.1186/1471-2458-6-164

**Published:** 2006-06-21

**Authors:** Sarah Wamala, Tony Blakely, June Atkinson

**Affiliations:** 1 Swedish National Institute of Public Health and Karolinska Institutet, Sweden; 2 Department of Public Health, Wellington School of Medicine, New Zealand

## Abstract

**Background:**

Both trends in socioeconomic inequalities in mortality, and cross-country comparisons, may give more information about the causes of health inequalities. We analysed trends in socioeconomic differentials by mortality from early 1980s to late 1990s, comparing Sweden with New Zealand.

**Methods:**

The New Zealand Census Mortality Study (NZCMS) consisting of over 2 million individuals and the Swedish Survey of Living Conditions (ULF) comprising over 100, 000 individuals were used for analyses. Education and household income were used as measures of socioeconomic position (SEP). The slope index of inequality (SII) was calculated to estimate absolute inequalities in mortality. Analyses were based on 3–5 year follow-up and limited to individuals aged 25–77 years. Age standardised mortality rates were calculated using the European population standard.

**Results:**

Absolute inequalities in mortality on average over the 1980s and 1990s for both men and women by education were similar in Sweden and New Zealand, but by income were greater in Sweden.

Comparing trends in absolute inequalities over the 1980s and 1990s, men's absolute inequalities by education decreased by 66% in Sweden and by 17% in New Zealand (p for trend <0.01 in both countries). Women's absolute inequalities by education decreased by 19% in Sweden (p = 0.03) and by 8% in New Zealand (p = 0.53). Men's absolute inequalities by income decreased by 51% in Sweden (p for trend = 0.06), but increased by 16% in New Zealand (p = 0.13). Women's absolute inequalities by income increased in both countries: 12% in Sweden (p = 0.03) and 21% in New Zealand (p = 0.04).

**Conclusion:**

Trends in socioeconomic inequalities in mortality were clearly most favourable for men in Sweden. Trends also seemed to be more favourable for men than women in New Zealand. Assuming the trends in male inequalities in Sweden were not a statistical chance finding, it is not clear what the substantive reason(s) was for the pronounced decrease. Further gender comparisons are required.

## Background

Historically, both New Zealand and Sweden have had a long tradition of universalism and welfarism, and targeted policies for equity. However, in the late 1980s and beginning of 1990s there was a substantial economic recession in both countries. New Zealand responded by considerable reduction in welfare and public services, as did Sweden although to a lesser degree [1-3]. Parallel to this phenomenon, socioeconomic inequalities in health have been shown to be increasing overtime in Sweden [4] and, in relative terms at least, in New Zealand too [5]. Likewise, relative inequalities in mortality have been trending upwards in other countries in Western Europe [6-8]. In one study, trends in mortality disparities between New Zealand and other Nordic countries (Finland, Norway and Denmark), with the exception of Sweden, was examined. The authors demonstrated that overall, relative inequalities in mortality widened equally rapidly in all four countries [9]. But it remains uninvestigated whether Sweden, with a strong history of egalitarianism, has had different trends in health inequalities.

The aim of this present study was to analyse trends in absolute socioeconomic inequalities in mortality in Sweden and New Zealand. We hypothesized that trends in socioeconomic differentials by mortality might be more favorable in Sweden compared to New Zealand. As it is already evident in the above discussion, the magnitude and trends in inequality vary depending on choice of absolute (e.g. rate differences in mortality between low and high socio-economic groups) or relative (e.g. rate ratios) measures. This issue has been an issue of debate in the past [10]. In this paper we have elected to focus mostly on absolute measures of inequality, although we also present relative measures.

## Methods

### Data sources

The New Zealand Census Mortality Study (NZCMS) was used for mortality analyses in New Zealand. The NZCMS comprise four cohorts formed by anonymous and probabilistic linkage of four censuses to 3 years of mortality records. The four cohorts were; early 1980s (1981–84) (all census respondents from 24^th ^March 1981); late 1980s (1986–89) (all census respondents from 4^th ^March 1986); early 1990s (1991–94) (all census respondents from 5^th ^March 1991); and late 1990s (1996–99) (all census respondents from 5^th ^March 1996). Detailed methods for linkages have been described earlier [11-13]. The NZCMS was approved by the Wellington Regional Ethics Committee (98/7) in compliance to the principles embodied in the Helsinki Declaration.

Similarly linked mortality data was obtained for Sweden using the Swedish Survey of Living Conditions (Undersökning för Levnadsföllhållanden- ULF). The ULF survey comprises a representative sample of the Swedish population between 16 and 84 years. Each individual participated in a one-hour face-to-face interview. In case a sampled person was not available, close relatives (spouse, parent or children) were interviewed instead. However this occurred in an insignificant number of sampled persons. These data comprise over 100, 000 men and women. Details about the survey are previously published elsewhere [14]. This survey data was linked to mortality data using routine registries.

The ULF survey linkage was agreed upon by the Statistics Sweden and the Swedish National Institute of Public Health (8336836/168603) in compliance to the principles embodied in the Helsinki Declaration.

We constructed four open cohorts (i.e. individuals were 'recruited' at each annual survey) and each cohort followed up for mortality for up to five years (i.e. all deaths up to the end of the follow-up period included). The following survey years were used; early 1980s (1980–85) (all live individuals interviewed on or after 4^th ^March 1980); late 1980s (1985–90) (all survey respondents interviewed on or after 4^th ^March 1985); early 1990s (1990–95); (all survey respondents interviewed on or after 5^th ^March 1990) and late 1990s (1995–2000) (all survey respondents interviewed on or after 5^th ^March 1995 and before 5^th ^March 2000). The last Swedish cohort was truncated at 31^st ^December 2001. We tried to make these cohorts comparable to those from the NZCMS but the dates are slightly different.

Socioeconomic position was measured using education and income. Both variables were classified into three-category variables, for descriptive presentation and calculation of slope of inequality (SII-see later in Methods). Education was classified for both Sweden and New Zealand according to the international OECD classification of education. The three-level categories were; i) low education (no qualifications, primary school 1 to 9 years of schooling), ii) medium (upper secondary school education), and iii) high (college or university education).

There are important differences in the income data between countries. In New Zealand, the measure was total income (including transfers and benefits, and before tax) self-reported on the census form using tick-box categories. The total personal income for all adults in the household was aggregated to get the total household income. By contrast, the Swedish income data for each ULF respondent was obtained by record linkage with the Taxation Office for the years 1980, 1985, 1990, 1995, respectively, for each cohort. This data was post-tax total income including earned income, government transfers and capital gains. These differences between countries mean that income was both more accurately collected in Sweden, and allowed for tax-transfers. The implication of this is that differences in mortality by income are likely to be bigger in Sweden than in New Zealand, simply due to better exposure measurement. Also, the Swedish income measure actually included a bit of asset wealth too, by virtue of including capital gains. In addition the Swedish income was categorised into tertiles based on the income distribution of each period, while that of New Zealand was based on 1986 income distribution.

On the Swedish data there was education information for almost every person, and those values that were missing were distributed over all the various years. Whereas for income, there was no Income values recorded for interview years 1979–1982 inclusive and this accounted for the majority of the missing income values. The remaining values that were missing were similar to the number of education values missing and had a similar distribution over the years.

Total household income was adjusted for inflation using the consumer price indices for both countries, but using 1980 as the base year in Sweden and 1996 in New Zealand. Household income was also equivalised for economies of scale. In New Zealand this was done using a New Zealand-specific equivalisation scale that adjusts for number of children and number of adults in the household [15]. In Sweden, a similar equivilisation scale of household income was used according to Statistics Sweden [16]. Income was categorised into approximate tertiles for both countries for descriptive analyses and for calculation of SIIs as with education above. We believe it is unlikely that different equivalisation methods will bias the comparisons of trends in this paper.

### Data analyses

Analyses were limited to respondents aged 25–77 years duringfollow-up period, i.e., we allowed aging in and aging out of the cohorts. Age standardisation mortality rates were calculated using the European population standard [17].

We used the slope index of inequality (SII) and relative index of inequality (RII) to measure absolute and relative, respectively, differences in mortality by income and education [18,19]. Briefly, these rate difference and rate ratio measures are calculated by ranking the population by the categories of socio-economic factor of interest. Each category is assigned a modified ridit score, equivalent to its mid-point on a cumulative proportion scale. For example, if the first group comprises 20% of the population it is assigned a value of (0.2/2 =) 0.1, and the second group comprises 30% of the population it is assigned a value of (0.2 + 0.3/2 =) 0.35, etc. The mortality rates for each category are then regressed on the modified ridit scores for each category, meaning that the beta or slope coefficient is the expected difference in mortality rates between the lowest (0^th ^percentile rank) and highest (100^th ^percentile rank) socio-economic positions in the population. This is the SII. The RII is calculated by dividing the expected mortality rate for the 0^th ^percentile by that for the 100^th ^percentile. The SII and RII have considerable advantages for cross-national comparisons, in particular, they are not prone to different group sizes and (somewhat) different categorisations of the socio-economic factor.

## Results

Table 1 demonstrates distributions of person years, education, income and number of deaths. In both Sweden and New Zealand, the proportion of men and women with low education has been decreasing, while increasing for high education (Table 1).

### Comparing rates

**Figure 1:**
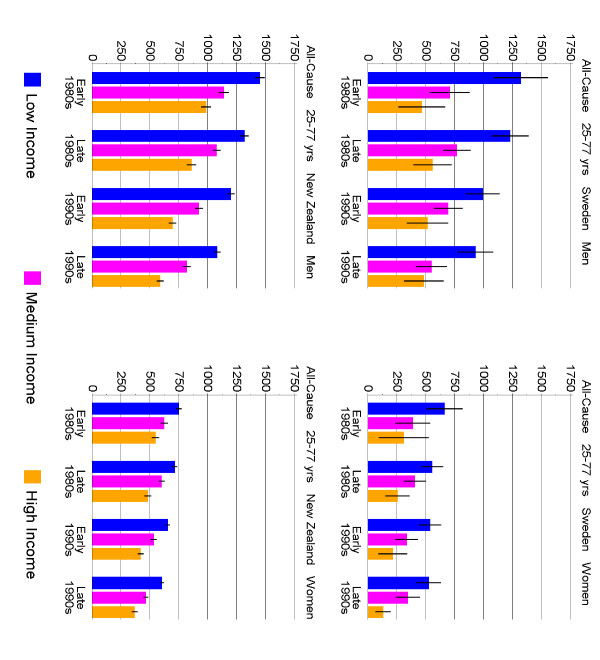
Mortality differentials by income (Standardised rates per 100 000).

**Figure 2:**
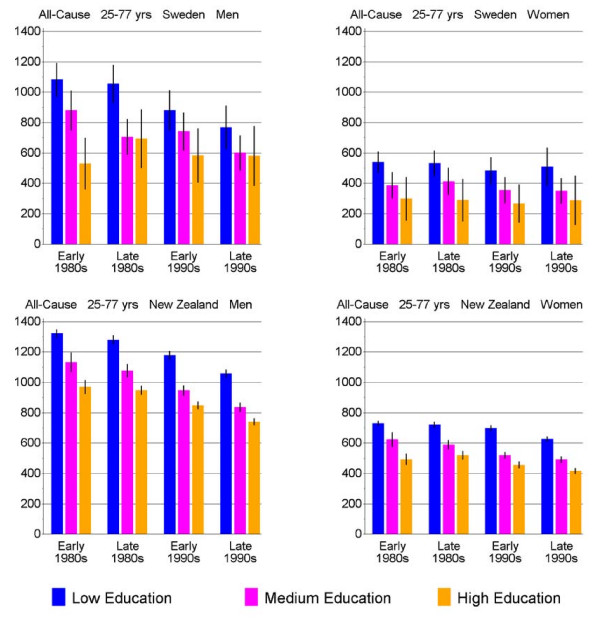
Mortality differentials by education (Standardised rates per 100 000).

Figures 1 and 2 show the standardized mortality rates by income and education, respectively. Although the mortality rates in Sweden were measured with much greater imprecision as reflected by the wide 95% confidence intervals, there was a clear pattern of higher mortality rates in lower socio-economic groups.

Consistent and statistically significant reductions in mortality rates were observed over time in all socio-economic groups in New Zealand for both sexes. Similar statistically significant reductions in Sweden were only observed among men with low income and women with high income – again probably a function of less statistical precision for Sweden. Of particular note, standardised mortality rates/100 000 among high-income women reached low levels in Sweden: the mortality rate decreased from 310 (95% CI 94–526) in early 1980s to 131 (95% CI 66–197) in late 1990s (p-trend = 0.01) (Figure 1).

### Comparing absolute inequalities in mortality, on average, over the 1980s and 1990s

A visual inspection of the absolute gaps in mortality rates between high and low socio-economic groups in Figures 1 and 2 suggests similar gaps in mortality by education between Sweden and New Zealand (with the exception of greater gaps among Swedish men in the 1980s). Gaps in mortality by income were clearly greater in Sweden. This is confirmed by an inspection of the SIIs shown in Table 3. (The SII is a regression-based estimate of the absolute difference in mortality rates between the lowest and highest socioeconomic group.) However, as suggested in the methods, we would expect greater differences in mortality by income in Sweden simply due to the better measurement of income (including some measurement of assets).

### Comparing trends in absolute inequalities in mortality over the 1980s and 1990s

The SIIs results in Table 3 quantify the visual impression of changing gaps in mortality rates across income and education shown in Figures 1 and 2. Included in Table 3 is a regression-based estimate of the percentage change in the SII from early 1980s to late 1990s, and p values for tests of trend. Swedish men clearly stand out as having different trends: a 51% decrease in the income SII (p-trend 0.06) and a 66% decrease in the education SII (p-trend < 0.01). Returning to Figures 1 and 2, the reasons for these pronounced trends are: notably high mortality for low socio-economic men in Sweden in the early 1980s compared to low mortality for high socio-economic males; and strong decreases in mortality among low socio-economic men compared to no real change in mortality for high socio-economic men. Whilst statistical imprecision of each of the men's Swedish mortality rates in the Figures is notable, the p for trend results were 0.06 and <0.01 for income and education, respectively.

For the three other groups (Swedish women, and New Zealand men and women), there were similarities in trends: approximately 10% to 20% increases in the income SIIs, and approximately 10% to 20% decreases in the education SIIs. Most trends in the SIIs were statistically significant or approaching statistical significance, with the exception of educational SIIs for women in New Zealand (8% decrease, p-trend 0.53). A closer inspection suggests possibly more favourable trends for men than women in New Zealand, consistent with the clearly more favourable trends for men than women in Sweden.

### Comparing trends in relative inequalities in mortality

The focus of this paper is on absolute inequalities in mortality, but for completeness we also present relative inequalities in Table 4. When average (regardless of socio-economic position) mortality rates are decreasing, trends in relative inequalities will appear worse than trends in absolute inequalities – but otherwise reflect patterns and trends in absolute inequalities. This is confirmed in Table 4. For example, whereas absolute inequalities in mortality by education decreased (Table 3), relative inequalities by education were stable or increasing with the exception of Swedish men. Likewise, trends in relative inequalities by income were more severe than trends in absolute inequalities by income. Of note, the RII becomes unstable when the estimated mortality rate for the highest socio-economic percentile (i.e. extrapolating beyond the midpoint of the highest socio-economic tertile) becomes close to zero – thus the very large income RIIs for Swedish women in late 1990s.

## Discussion

Consistent and statistically significant reductions in mortality rates over time were observed in all socio-economic groups in New Zealand, while similar reductions in Sweden were only observed among men with low income and women with high income. Regarding absolute inequalities in mortality *on average *over the 1980s and 1990s, they were similar between Sweden and New Zealand by education for both men and women (with the exception of greater inequalities among men in Sweden in the 1980s). Absolute inequalities in mortality by income were greater in Sweden, although this is almost certainly due to better income measurement in Sweden. Regarding *trends *in absolute inequalities, there was a strong decreasing trend for men in Sweden (66% by education and 51% by income). For both men and women in New Zealand, and women in Sweden, there were approximately 12% to 21% increases in inequality by *income *and 8% to 19% decreases in inequality by *education*. Trends were clearly most favourable for men in Sweden, and possibly also more favourable for males in New Zealand.

The results presented in the present study should be interpreted with awareness of potential limitations. First the New Zealand data base was larger than that of Sweden which makes it difficult to draw conclusions in the presence of wide confidence intervals. However, many of the statistical tests of trend were significant. In addition, in spite of relatively smaller population for Sweden, ULF is a random sample representative of the Swedish population. In addition Swedish mortality rates per 100,000 by socio-economic position were comparable to the national rates (with reservations for varying age groups and standardisation methods) [20].

Second, sources of income data varied in the two countries. Due to the taxation system, Sweden has better income measurement than New Zealand, such that the inequalities by income in Sweden probably shift up relative to New Zealand. Thus some, if not all, of the mortality differentials by income may almost certainly be due to methodological aspects. It seems likely that mortality disparities by income in Sweden are 'too well captured' to be comparable to countries such as New Zealand at any one point in time. However, comparisons of trends over time are likely to be valid, so long as varying baselines are allowed for.

Furthermore, income was measured at the household level and didn't distinguish women's from men's individual income. This may make it difficult to draw conclusions on the observed gender differences. However, a study by Fritzell at al showed that health effects were similar regardless of whether household or individual income was used [21]. There are also conceptual advantages with household income as opposed to individual income as a measure of one's ability to purchase items.

The advantage of this study is that it provided us with the opportunity to study trends in socioeconomic inequalities in mortality, using both education and income, among men and women. This is the first study we are aware of where inequalities are investigated from a gender perspective comparing Sweden with another non-European country, over a long period of time.

There are no strictly comparable published Swedish studies on trends in socioeconomic inequalities in mortality. Trends in total mortality by socioeconomic status have often been limited to younger population up to 64 years [20] and to specific causes of death. Gender differences in socioeconomic differentials have often been interpreted as being less among younger women than among younger men, but this is in part due to lower overall mortality rates among women than men. Because of this, absolute differences in mortality rates between low and high socio-economic groups are greater among men (but may be similar or greater in relative terms among women – as shown in the present paper.

In fact in a previous comparison of absolute mortality rates by occupational class between late 1980s and early 1990s demonstrated that mortality had decreased among men and women (aged 20 to 64 years) across all occupational classes with an exception of women with blue collar jobs [20]. Reinterpreting previous comparisons in this light, trends in socioeconomic differentials in women's mortality are expected to be decreasing on a slower rate than those for men – as demonstrated in the present study.

Trends in absolute (and relative) inequality by education and income in New Zealand have been published before [22]. These previous results adjusted for ethnicity (a confounder of the association of socio-economic position with mortality in New Zealand), but the trends over time in inequalities were similar to those published in this current paper.

We found trends in socioeconomic inequalities in mortality among women to be similar in Sweden and in New Zealand data. Results in the present study suggest that women have not benefited as much as men from the reduction in socioeconomic inequalities in mortality in the past 20 years, especially in Sweden. That said, men's inequalities by education in Sweden appeared to start at a very high level in the early 1980s, but decreased markedly to smaller inequalities than those for men in New Zealand and for women in Sweden, when measured by education. Whilst a statistical chance might explain men's trends in Sweden, (although tests of trend were statistically significant or nearly so) at least two substantive reasons might explain this divergence in trends between men and women in Sweden. Measures of SES for women, particularly income, have become better (relative to men) in recent years due to increased participation in the labour market, which may show more inequalities than in the past – or at least cause increasing income-related inequalities to be observed among women despite decreases among men.

Another potential explanation, but one that we do not think is likely, is the changing proportion of single women. In Sweden for example, during the 1990s there was an increase in the proportion of single parents (about 20% of adults, and of them 70% were women) [23]. Single parenting has been associated with economic hardships [24] and increased mortality [25]. Whilst both Sweden and New Zealand have welfare benefits specifically for solo parents, they are probably not sufficient to maintain the same level of equivalised household income as before any separation from an income-earning partner. However, single parents seem an unlikely driver of the results we see, for two reasons: mortality among 25–77 year olds is driven by adults older than those with dependent children; and whilst a greater portion of low income households may now be made up of single parent households, it does not necessarily follow that the mortality rate differences between low and high income thus will also increase.

We are not sure of the reasons for a possible profound declining trend in absolute gaps in men's mortality by socioeconomic position. However, the Swedish trends in ischemic heart disease (IHD) mortality, which is a major contributor to total mortality, may in part explain the observed declining trends. Rosengren et al have shown a larger decrease in cardiovascular morbidity among men than women between 1984 and 1999 [26]. In addition Hallqvist et al demonstrated that a decline in mortality due to myocardial infarction (MI) among men in high socioeconomic position men started in the 1970s while that of men in low socioeconomic position started in early 1980s [27]. Thus it is possible that the rapid decline in mortality due to MI occurred first in high socio-economic men (say in the 1970s), and later in lower socio-economic men (say in the 1980s and 1990s). If true, this would mean that our study of the 1980s and 1990s would have missed the rapid fall among higher socio-economic men (and consequent widening absolute gaps), and just observed the 'correction' as men in lower socio-economic position caught up. Such dynamic trends have been proposed by Victora as a result of the inverse equity hypothesis [28]. Regardless, the dynamic nature of trends in inequalities over time is something that both scientists and policy makers must increasingly consider and try to understand.

Why might trends in absolute inequalities by education be decreasing, but by income increasing? First, we discuss above that increasing participation by women in the labour market may explain their increasing inequalities by income. Regarding declines in absolute educational inequalities, for both men and women, one possible reason is that there is a shift in western industrialised societies for income being a greater axis of stratification than education (other than education influencing later income), which may explain why educational gaps are tending to decrease and income gaps are tending to increase. Second, it is possible that education is becoming a weaker marker of socio-economic stratification particularly in Sweden due to the fact that that there are increasingly fewer people with no qualifications. However, the SII and RII methods used in the present paper deal with this problem. Third, and more simplistically, it may just be a mathematical consequence of *absolute *inequalities having to decrease at some point when average or background mortality rates are relentless falling (although *relative *inequalities may continue to widen, eg Table 4 of this paper).

Both New Zealand and Sweden have current national strategies to tackle health inequalities. New Zealand's strategy was established about 5 years ago [29], while Sweden has had a long history to tackle health inequalities. In fact, Sweden is the first country to endorse a unique national public health policy which was agreed on by a majority of political parties with the intention to promote good health for all [30,31]. Based on the results of the present study, these strategies seem to (somehow) have been outstandingly successful for men. It is yet to be seen if these strategies will in the long-run also contribute to more successful reductions in socioeconomic inequalities among women's mortality.

## Conclusion

Trends in socioeconomic inequalities in mortality were clearly most favourable for men in Sweden. These trends may also have been more favourable for men than women in New Zealand. Assuming the trends in male inequalities in Sweden were not a statistical chance finding, it is not clear what the substantive reason(s) was for the pronounced decrease. Thus further gender comparisons are required.

## Competing interests

The author(s) declare that they have no competing interests.

## Authors' contributions

Sarah Wamala contributed to the generation of hypotheses and the conceptualization of the study and led the writing of the manuscript. Tony Blakely supervised the analyses and contributed to the interpretation of the findings and to the writing of the manuscript. June Atkinson contributed to the synthesizing of analyses and carried out statistical analyses. All authors helped to conceptualize ideas, interpret findings, and review drafts of the manuscript.

## Pre-publication history

The pre-publication history for this paper can be accessed here:



## Figures and Tables

**Table 1:** Distributions by education, age 25–77 years

**Sex**	**Country**	**Time period**	**Person year at risk 1000s**	**No. of Deaths**	**Low education %**	**Medium education %**	**High education %**
Men	Sweden	1980–85	129	672	40	41	19
		1985–90	115	561	35	43	22
		1990–95	110	423	28	45	27
		1995–00	89	291	24	48	28
							
	New Zealand	1981–84	4480	22194	52	18	30
		1986–89	5057	22578	37	19	44
		1991–94	5584	22842	33	21	47
		1996–99	5944	22056	32	25	42
							
Women	Sweden	1980–85	132	387	45	38	17
		1985–90	118	324	37	41	22
		1990–95	113	252	28	45	27
		1995–00	93	195	24	47	29
							
	New Zealand	1981–84	4491	13761	59	19	21
		1986–89	5073	14118	46	24	30
		1991–94	5780	14631	37	28	35
		1996–99	6258	14436	34	32	34

**Table 2:** Distributions by income, age 25–77 years

**Sex period**	**Country**	**Time**	**Person time (1000s)**	**No. of deaths**	**Low income %**	**Medium income %**	**High income %**	Median income*
Men	Sweden	1980–85	52	270	32	34	33	141700
		1985–90	114	564	32	34	34	229440
		1990–95	109	420	33	34	34	211400
		1995–00	88	288	33	34	33	231100
								
	New Zealand	1981–84	3845	19203	28	31	41	37200
		1986–89	4381	20781	29	34	37	36000
		1991–94	4659	19545	31	29	41	37500
		1996–99	4938	17958	30	27	43	38800
								
Women	Sweden	1980–85	53	162	34	32	33	138990
		1985–90	117	321	35	32	33	225670
		1990–95	111	249	34	33	33	209700
		1995–00	92	192	33	33	34	231100
								
	New Zealand	1981–84	4028	11979	36	30	34	33400
		1986–89	4589	13251	36	33	31	32600
		1991–94	4938	12597	38	27	35	32400
		1996–99	5248	11553	36	26	38	34600

*Median income: for Sweden in Swedish kronas (SEK) and for New Zealand in NZ dollars (NZD). 1 NZD is approximately equivalent to 6 SEK

**Table 3:** Absolute inequality (SII) for all cause mortality 25–77 year olds by income and education

**By income**							
		**Early 1980s**	**Late 1980s**	**Early 1990s**	**Late 1990s**	**% change†**	**P-trend ‡**

**Men**							
All-Cause	Sweden	1265 (598–1932)	(546–1445)	716 (503–929)	669 (138–1200)	↓ 51%	0.06
	New Zealand	679 (344–1015)	677 (593–761)	779 (638–919)	766 (635–896)	↑ 16%	0.13
**Women**							
All-Cause	Sweden	535 (207–862)	453 (451–455)	485 (346–625)	589 (524–655)	↑ 12%	0.03
	New Zealand	308 (234–382)	347 (321–374)	369 (365–373)	374 (307–441)	↑ 21%	0.04

**By education**							

**Men**							
All-Cause	Sweden	719 (336–1103)	560 (16–1104)	409 (371–446)	243 (-38–524)	↓ 66%	<.01
	New Zealand	598 (542–655)	558 (428–688)	530 (280–780)	496 (262–731)	↓ 17%	<.01
**Women**							
All-Cause	Sweden	354 (331–377)	339 (285–393)	299 (230–369)	294 (105–483)	↓ 19%	0.03
	New Zealand	370 (228–512)	333 (281–384)	380 (202–558)	320 (223–417)	↓ 8%	0.53

† The percentage change is from 1981–84 to 1996–99, estimated by fitting a ordinary least squares regression (unweighted) to the SIIs to work out the regression-estimated change in the SII over time, the regression estimated value for 1981–84, and hence the percentage change.

‡ We conducted ordinary least squares regression of the SII on census year (weighted by the inverse of the variance of the SII), and used the p-value for the census year term as our p-value for trend.

**Table 4:** Relative inequality (RII) for all cause mortality, 25–77 years

**By income**						
**Relative Index of Inequality (RII)**						
	**Income**	**Early 1980s**	**Late 1980s**	**Early 1990s**	**Late 1990s**	**P-trend**
**Men**						
All-Cause	Sweden	7.75 (1.90–31.59)	3.88 (2.13–7.08)	2.92 (1.61–5.28)	3.08 (1.54–6.17)	0.22
	New Zealand	1.82 (1.67–1.98)	1.93 (1.78–2.09)	2.47 (2.26–2.71)	2.83 (2.56–3.14)	0.03
						
**Women**						
All-Cause	Sweden	3.83 (1.03–14.27)	3.50 (1.62–7.59)	4.98 (1.54–16.11)	14.9 (0.9–36.35)	0.28
	New Zealand	1.63 (1.47–1.80)	1.80 (1.64–1.99)	2.04 (1.84–2.25)	2.28 (2.0–2.54)	<.01

**By Education**						

**Men**						
All-Cause	Sweden	2.35 (1.59–3.47)	2.03 (1.34–3.08)	1.77 (1.12–2.78)	1.47 (0.87–2.50)	<.01
	New Zealand	1.68 (1.55–1.82)	1.68 (1.57–1.80)	1.75 (1.63–1.87)	1.80 (1.68–1.93)	0.04
						
**Women**						
All-Cause	Sweden	2.35 (1.35–4.07)	2.30 (1.26–4.22)	2.37 (1.17–4.82)	2.31 (0.98–5.43)	0.91
	New Zealand	1.78 (1.59–1.99)	1.72 (1.57–1.88)	2.02 (1.85–2.20)	1.91 (1.75–2.07)	0.35
